# Effects of a forced cycling program with cognitive stimulation on symptomatology, physical condition, and cognition in people diagnosed with Parkinson disease

**DOI:** 10.1097/MD.0000000000031920

**Published:** 2022-12-02

**Authors:** Karina Pitombeira Pereira-Pedro, Iris Machado de Oliveira, Irimia Mollinedo Cardalda, José M. Cancela-Carral

**Affiliations:** a Department of Special Didactics, Faculty of Education and Sports Sciences, HealthyFit Research Group, University of Vigo; b Department of Functional Biology and Health Sciences, Faculty of Physiotherapy, Healthy Fit Research Group, University of Vigo.

**Keywords:** cognitive-motor interference, cycling, dual-task, Parkinson disease

## Abstract

Parkinson disease may present difficulties in performing dual tasks. The use of dual tasks during training can improve different abilities. Therefore, the objective of this study is creating a protocol, based on standard protocol items recommendations for interventional trials compliant, for a clinical trial study conduct, review, reporting, and interpretation. This protocol will provide the framework and a guide to a randomized double-blind study, that will be conducted to assess the influence of a cycling exercise program combined with a cognitive task, on cognitive and physical Parkinson disease aspects.

**Methods:**

Designed a protocol for a double-blind randomized study, where participants will perform a dual tasks intervention with cycling and a cognitive task. The revised version of the unified Parkinson disease rating scale, the Parkinson disease questionnaire, the timed up and go Test, the 30 seconds chair sit to stand test, the Stroop and the trail making test will be used to measure outcomes.

**Discussion:**

Research in Parkinson disease suggests that an improvement of motor and cognitive functions of Parkinson disease patients can be achieved by modifying different motor and cognitive pathways. The results of the present study will yield findings on both the physical and cognitive response to an intervention that combines a cognitive task with a motor task in Parkinson disease patients and will be essential tool for a better conducting of the clinical trial study.

## 1. Introduction

Parkinson disease (PD) is a degenerative neurological disease that causes motor impairments such as difficulty in task switching, functional walking, and postural control. In addition, cognitive impairment in PD also includes impairments in executive function, attention, memory, cognitive and sensorimotor processing. These may therefore lead to difficulties in complex activities, such as dual tasks.^[1]^

A dual task (DT) is the simultaneous execution of 2 tasks, where 2 concurrent tasks compete for limited resources, inducing dual-task interference and deterioration in the performance of one or both tasks.^[2]^ The performance of DT depends on the ability to perform motor tasks automatically and the cognitive ability to combine different types of tasks.^[3]^ The population with PD may present difficulties in performing dual tasks, for example in combining physical and cognitive tasks. The dopamine deficiency in the basal ganglia loops causes the integration of cognitive and motor information to be impaired. The limited processing of motor-cognitive information in patients with PD results, therefore, in inadequate responses to stimuli during movement in complex everyday situations and dual-task situations.^[4]^

In the parkinsonian population, physical exercise and exercise therapy have been shown to have positive effects on the improvement of conditioned physical abilities such as gait, balance, coordination, and mood, thus improving the quality of life of these individuals.^[5]^ Forced cycling interventions have shown improvement in motor and non-motor symptoms in individuals with PD.^[6,7]^ This type of exercise makes the individuals actively participate by performing revolutions of their lower limbs while assisted by the cycling device, leading to potential improvements in the locomotor system, which has been affected by the impaired functioning of the central nervous system.^[8]^ The hypothesized key mechanism of forced cycling interventions is that they are performed at a high cadence. A cadence of 80 revolutions per minute (rpm) has been found to produce neuroprotective results and improved motor function.^[9]^

Recent review studies have explored the effects of DT in PD patients and proposed that the use of DT during training can improve gait performance, balance ability and other motor skills in PD patients.^[10,11]^ However, studies that analyze the effect of DT on both aspects, cognitive dysfunction associated with the physical aspects of the PD, such as disease symptomatology, quality of life, dynamic balance, strength, and heart frequency, are not easily found. Therefore, the objective of this study is to assess the influence of a cycling exercise program combined with the performance of a cognitive task, on cognitive and physical aspects in PD patients. To carry out this investigation, we will carry out a double-blind, randomized study, with parallel groups, a 1:1 allocation rate and an exploratory framework.

## 2. Methods

### 2.1. Design

A double-blind, randomized study. It will be conducted at the Laboratory of XXXXXXX, in the Faculty XXXXXXXXXXX. Participants will be recruited from a population of patients with PD treated in the “XXXXXXXXXX” - Galicia, Spain. They will be randomly allocated (1:1) to an experimental group (EG) or a control group (CG). Outcome measures will be collected by trained researchers at the start (week 0) and at the end of the intervention (week 12). This project was approved by the Research Ethics Committee of the “XXXXXXXXXXX” 205-2022-2, under protocol number and registered as a clinical trial at www.ClinicalTrials.gov (ID: XXXXXXXXX).

The present protocol has been prepared in accordance with relevant items from the standard protocol items: recommendations for interventional trials Checklist (see Additional file 1) and the standard protocol items recommendations for interventional trials Table (Table 1).

**Table 1:** The schedule of enrollment, interventions, and assessments in the Standard Protocol Items: Recommendations for Interventional Trials (SPIRIT).

	Study period					
	Enrolment	Allocation	Post-allocation			
Time point	0	0	1 wk	4 wk	8 wk	12 wk
**Enrolment**						
Eligibty screen	X					
Informed consent	X					
Allocation		X				
**Interventions**						
Control group (30 min. cycling)			X	X	X	X
Experimental group (30 min. cycling combined with cognitive task)			X	X	X	X
**Assessments**						
MDS-UPDRS			X			X
PDQ-39			X			X
TUG			X			X
Cognitve tests (stroop and TMT A‐B)			X			X
Chair SIT-TO-stand test			X			X
Muscle parameters			X			X

MDS-UPDRS = movement disorder society´s unified Parkinson´s disease rating scale, PDQ-39 = Parkinson´s disease quality of life questionnaire, SPIRIT = standard protocol items recommendations for interventional trials, STROOP = Stroop color-word test, TMT A-B = trail making test (part A and B), TUG = timed up and go test.

### 2.2. Participants

Will be recruited patients of both sexes diagnosed with idiopathic PD, who are members of the “XXXX” and who met the following inclusion criteria will be recruited: being diagnosed with idiopathic PD of a stage within 1 to 2 Hoehn and Yahr scale; no clinical history of dementia, neurological deficits, or any other preexisting condition that might limit limb movement; no history of medical or surgical intervention that could interfere with motor function; and no medical contraindication for performing therapeutic exercises.

In this study, the ethical standards outlined by the Declaration of Helsinki will be followed, and all the participants will be informed and will give their written consent prior to participating in the study.

A random number will be assigned to each of the participants and only the principal investigator will have access to the coding system. Data will be recorded in computer support, in a database created for this project and that complies with the *Ley Orgánica de Protección de Datos* 15/1999.

### 2.3. Intervention

The MOTOmed Viva 2 Parkinson System movement therapy device created by the RECK Company will be used to perform the intervention. It is an intelligent ergometric bicycle, with motorized drive mechanisms with which non ambulatory or disabled patients can move their legs repeatedly, assisted or forced, while sitting in their wheelchair or in a regular chair.^[12]^

We will use a cycling program with 30 minutes of intervention, where there will be 6 minutes of passive cycling as a warm-up (2 minutes 40 rpm, 2 minutes 70 rpm, and 2 minutes 90 rpm), 20 minutes of active-assisted cycling (90 rpm) and 4 minutes passive cycling to cool down (40 rpm). The sessions will be undertaken twice a week, always leaving at least 2 days of rest between the 2 sessions. The duration of the program will be 24 sessions, each session being 30 minutes long.

The assessment of the disease symptomatology, quality of life, cognitive function, dynamic balance, strength, and heart frequency will be carried out twice: first, before of starting the intervention (pre-intervention), and the second time after the last session (post-intervention). The assessments will be carried out by a physiotherapist who will not be involved in the intervention.

Failure to comply with at least 80% attendance of the intervention will lead to the exclusion of the participants from the results analysis. In addition, to improve adherence to the intervention, alternative days will be established for carrying out the sessions, always respecting a period of 2 days’ rest between sessions.

### 2.4. EG and CG protocol

Both groups will be participating in the intervention and completing 2 sessions per week for 12 weeks. Both will undertake the 30 minutes exercise program using the MOTOmed Viva 2 Parkinson system. The difference between them will be that the EG will combined the cycling with a cognitive task.

The cognitive task will be a PowerPoint presentation structured in a coherent way alongside the phases of the cycling program. The passive phases (6 minutes warm up and 4 minutes cool down passive cycling), will spent be looking at images of a familiar, nearby environment.

For the active phase (20 minutes cycling active assisted) PowerPoint presentation will be structured with tasks related to:

(1) Orientation (composed of 5 tasks, for example: temporal orientation, spatial orientation)
(2) Memory (composed of 3 tasks, for example: memorize objects/animals)
(3) Calculation (composed of 10 tasks, for example, geometry, addition, subtraction and multiplication)
(4) Language (composed of 6 tasks, for example: spelling backwards, ordering sentences)
(5) Similarities (composed of 4 tasks, for example: group foods of the same color).

**Figure 1.**
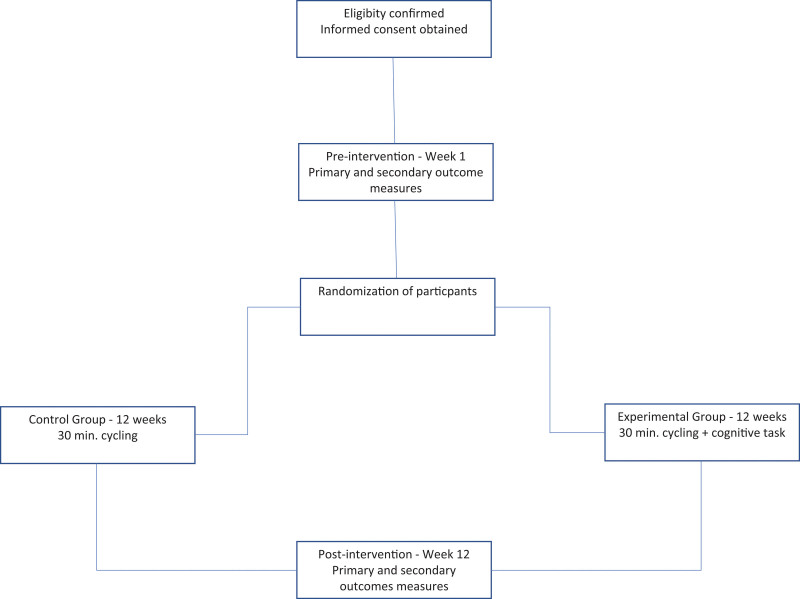
Schematic study design.

The participants will have to pay attention to the tasks and think about the answers. In each session different task contents will be presented, there will be a total of 30 different cognitive tasks (considering the sequence of images projected in the heating and cooling phase). The schematic study design is shown in next figure (Fig. 1).

### 2.5. Outcomes

#### 2.5.1. *Primary outcomes*.

The PD symptomatology will be assessed using the adapted Spanish version of the movement disorder society unified Parkinson disease rating scale (MDS-UPDRS).^[13]^ The MDS-UPDRS total score range is from 0 to 200, with higher values indicating a greater impact of PD symptoms. It should be noted that this scale consists of 4 parts: I, nonmotor aspects of daily life; II, motor aspects of daily life; III, motor aspects of the disease; and IV, dyskinesia.

For the evaluation of the quality of life, the Spanish version of the Parkinson´s disease quality of life questionnaire will be used, validated in Spanish by Martínez-Martín, Frades Payo and “The movement disorders society study groups.”^[14]^ This questionnaire consists of 39 items with 5 response alternatives (0 = never, 1 = occasionally, 2 = sometimes, 3 = frequently, 4 = always/unable), which make up 8 dimensions: mobility, activities of daily living, emotional well-being, stigma, social support, cognitive impairment, communication, and bodily discomfort.^[15]^ Each domain is expressed as a percentage on the maximum score of the same, so that, the higher the score, the greater the deterioration of the quality of life.

Dynamic balance will be evaluated by means of the timed up and go (TUG) test, which consists of getting up from a chair with armrests, walking 3m toward a cone, turning around the cone, and returning to the starting position and sitting down again in the shortest possible time. This test is used primarily as a measure of mobility but is also useful as a measure of bradykinesia while walking.^[16]^ The test will be performed 3 times: walking normally; walking whilst saying names of animals and walking whilst reciting a mental calculation (30-3 = 27, 24, 21...).^[17,18]^ On the 3 conditions, the TUG test will be undertaken using Wiva scientific sensor wireless inertial detection devices set between the L4 and L5 vertebrae (Let Sense Group, Castel Maggiore, Italy).

Lower limb strength will be evaluated by the 30 seconds chair sit to stand test, belonging to the Senior Fitness test battery created by Rikli and Jones.^[19]^ This test consists of getting up from and sitting down on a chair as many times as possible in 30 seconds. The arms should be crossed over the chest, and the person should sit down fully, transferring their weight to the chair, and rise fully, to hip extension.

#### 2.5.2. *Secondary outcomes*.

The Stroop Colour-Word Test will be used to measure mental speed and response inhibition. In this test the subjects are required to name the ink color in which a word stimulus is printed, and the level of conflict is manipulated by varying the task-irrelevant property of the stimuli (in this case the words’ meanings), from conflicting or “incongruent” (e.g., the word “Red” printed in green ink) to no conflicting neutral, or “congruent” properties (e.g., the word “Red” printed in red ink). The test will be performed with 3 sheets, the patient having 45 seconds to perform the task printed on each sheet. On the first sheet, 3 words will be written arbitrarily in black ink: red, blue and green, and the patient simply will have to read them, the number of correct answers made in this way will be identified as words (W). The second card will be made up of coloured symbols of red, blue, and green; the patient will have to identify the colors, with the number of correct answers in this form will be identified as colors (C). Based on the results obtained in these first 2 sheets, a mathematical formula will be used to calculate the Colours-Words Planned, which would be considered the number of correct answers that the patient should get right on the third sheet (P × C/P + C = Colours-Words Planned). Finally, on the last sheet, the intention will be that the patient was able to read each word without making mistakes, while considering that the written terms the names of colors were written in colors that do not correspond to their original meaning. The result of the test will be the interference calculated by the difference between Colours-Words Planned and the result obtained in the last sheet.^[20]^

The trail making test (TMT) (part A and B) will be used to assess the mental speed and cognitive flexibility, respectively. TMT-A requires individuals to connect 25 targets on a computer screen by joining numbers in sequential order, and TMT-B requires alternating between 25 lettered and numbered targets. The score on each of these tests represents the amount of time it takes to complete it.^[21]^

The MOTOmed Viva 2 Parkinson will be used to carry out the patients’ exercise program. The system stores data on cardiac frequency, muscle tone, symmetry, coordination, and spasms for each of the sessions undertaken.

### 2.6. *Sample size estimates*

Sample size calculation will be done using the statistical power analysis program G*Power 3.1, which allows this type of analysis to be carried out in studies designed with an intergroup variable and repeated measures. The calculation will be carried out based on the results obtained in 2017 by Carroll et al^[22]^ on the effect of exercise on motor symptoms MDS-UPDRS. We will consider a percentage of 10% of expected losses, a level of confidence or security (1-*α*) of 95% and a statistical power (1-*β*) of 0.95, which allows to define a sample size of 44 people (22 per group). A stratified randomization process will be performed by an external investigator, considering the Hoehn & Yahr stages.

### 2.7. Randomization

Randomization will be computer-generated in randomized blocks using the randomization.com system. The process will be carried out by a volunteer not affiliated with the research, who will preserve allocation anonymity, randomly separating the individuals into a CG and EG the volunteer will also prepare sealed envelopes, using codes to represent the groups.

Only the researchers in charge of conducting the training sessions will be aware of meaning of each code and participant allocation. They will open the envelope corresponding to each patient number before the patients start the training.

The researchers responsible for the initial assessment and reassessment will not be informed of allocation during data collection and statistical analysis. Data analysis will be performed by a person who did not participate in the intervention, and they will only have access to the coding and will not be informed to which group each code corresponds.

### 2.8. Data analysis

To determine the effect of the intervention program on the symptomatologic variables MDS-UPDRS, quality of life Parkinson´s disease quality of life questionnaire, cognitive function (Stroop and TMA A-B), dynamic balance (TUG test), muscular parameters (Chair sit-to-stand, tone, symmetry, coordination, and spasms) and heart frequency in PD, a comparative study (pre-post) will be carried out, defining the significant statistical difference (p 0,05) in each of the parameters. A statistical analysis will be carried out of the results obtained as a group on the variables that were characterized by the intervention program (number of sessions, work, speed, and distance). Statistical calculations will be performed by using IBM SPSS 25.0 computer software.

## 3. Discussion

The protocol aims to verify the influence of a cycling exercise program combined with the performance of a cognitive task, on cognitive and physical aspects in PD patients. This protocol was based on our pilot study^[23]^ with little alterations that add a more aerobic nature and time of the intervention. In this pilot study, there was a detrimental to the performance of cycling activity combined with a cognitive task, and generated the reflection that the idea of combining cycling with a cognitive task in a supposedly safer environment, such as using a stationary bicycle, may have benefits for patients with PD. Furthermore, on a cognitive level, an improvement trend was observed in the group that performed the cognitive training, which can also be considered a positive point, since for the DT the physical and cognitive factors can be equally important.

In our protocol, 30 minutes of active/assisted passive cycling intervention will be carried out, structured so that the first 6 minutes are passive cycling for the initial warm-up (going from minute 1 of 40 rpm until reaching 90 rpm at the end of minute 6), followed by 20 min of assisted active assisted cycling at 90 rpm and finally ending with 4 min of passive cycling for cool down at 40 rpm. The cognitive task will consist of a structured on-screen slide presentation at the same time as the cycling phases. In the passive phases, images of the environment close to the patients will be presented and in the active phase a series of tasks will be undertaken that include aspects of orientation, memory, calculation, language, and similarities.

The degenerative nature of Parkinson disease leads to a progressive deterioration of different motor skills (this being what the disease is best known for) but also a progressive deterioration of sensory and cognitive capacity^[24–27]^ that are still key pieces in the structuring of a correctly performed motor task. Patients with PD have difficulty performing motor tasks and their ability to perform 2 tasks simultaneously can present significant alterations. In the early stages of PD, the ability to perform automatic tasks may also be impaired. The evidence indicates that the alteration of the sensorimotor Striatum in patients with PD prevents automatic control, and that patients with PD require more attentional control to perform everyday tasks.^[27,28]^

In addition, in relation to the cognitive alteration present in patients with PD, these can present themselves in a very varied way. In fact, such variability may be due to different, and sometimes overlapping, neurobiological mechanisms. Furthermore, some specific domains of cognition may be more affected than others.^[29]^ There is evidence that specific cognitive domains such as attention and executive function contribute to better gait development and general mobility in neurodegenerative processes.^[26]^

Different studies have found benefits in different motor aspects for Parkinson patients from interventions based, for example, on cycling with Parkinson patients.^[25]^ Research in PD suggests that an improvement of motor and cognitive functions of PD patients can be achieved by modifying different motor and cognitive pathways.^[27]^ Hsiu-Chen et al^[28]^ observed in their study that the facilitation or interference of the Dual task in both motor and cognitive performance can be influenced by the type of motor task that is carried out. In their study, they concluded that the combination of cycling with a cognitive task has a facilitating effect on cognition, and they propose it as a potential adjunctive treatment strategy to improve motor and cognitive functions in patients with PD. The question now lies in how the association of the motor task with the cognitive task influences both aspects simultaneously, rather than each one separately.

One strength of the current protocol is precisely this combination of the motor task and the cognitive task. However, in this study, the probable lack of homogeneity in the sample may result in different starting points both at a functional and cognitive level. Starting from different situations and the possible combinations that result, generates a weakness when it comes to extrapolating the results.

The results of the present study will yield findings on both the physical and cognitive response to an intervention that combines a cognitive task with a motor task in PD patients. Such results will create the base from which to work and will provide elucidations of how we should include a cognitive task associated with a motor task and which potential limitations of such an intervention would need modifying based on the responses of the patients.

## Acknowledgements

Parkinson XXX Association and the families of the participants who will allow the study to be carried out.

## Author contributions

**Conceptualization:** Karina Pitombeira Pereira-Pedro, Iris Machado de Oliveira, Irimia Mollinedo Cardalda.

**Investigation:** Karina Pitombeira Pereira-Pedro, Iris Machado de Oliveira, Irimia Mollinedo Cardalda.

**Methodology:** Karina Pitombeira Pereira-Pedro, Iris Machado de Oliveira, Irimia Mollinedo Cardalda.

**Project administration:** Iris Machado de Oliveira.

**Supervision:** Iris Machado de Oliveira, José M. Cancela-Carral.

**Visualization:** José M. Cancela-Carral.

**Writing – original draft:** Karina Pitombeira Pereira-Pedro, Iris Machado de Oliveira, Irimia Mollinedo Cardalda, José M. Cancela-Carral.

**Writing – review & editing:** Karina Pitombeira Pereira-Pedro, Iris Machado de Oliveira, Irimia Mollinedo Cardalda, José M. Cancela-Carral.
